# Urine shedding patterns of pathogenic *Leptospira* spp*.* in dairy cows

**DOI:** 10.1186/s13567-023-01190-w

**Published:** 2023-07-31

**Authors:** Gustavo Monti, Victor Montes, Pablo Tortosa, Carlos Tejeda, Miguel Salgado

**Affiliations:** 1 Quantitative Veterinary Epidemiology Group, Wageningen University and Research, Wageningen, The Netherlands; 2 Programa de Doctorado en Ciencias Veterinarias, Escuela de Graduados Facultad de Ciencias Veterinarias, Universidad Austral de Chile, Valdivia, Chile; 3 Department of Veterinary, Faculty of Veterinary Sciences, Universidad Técnica de Manabí, Portoviejo, Ecuador; 4 UMR PIMIT Processus Infectieux en Milieu Insulaire Tropical, Université de La Réunion, Ste Clotilde, Ile de La Réunion, France; 5 Instituto de Medicina Preventiva Veterinaria, Facultad de Ciencias Veterinarias, Universidad Austral de Chile, Valdivia, Chile

**Keywords:** Leptospirosis, urine shedding, cattle, transmission, dairy cows

## Abstract

Pathogenic *Leptospira* spp. are zoonotic bacteria that infect wild and domestic animals. Humans contract leptospirosis directly through contact with infected animals or indirectly from contaminated water or soil. In mammalian reservoirs, the pathogen can colonize renal tubules for lengthy periods and persistently contaminate the environment through urine. Cattle have been reported to shed several serovars; with Hardjo the most common serovar found in cattle. Without clinical manifestations, the infection can spread within a herd, impairing productivity, and putting workers like farmers, abattoir operators and veterinarians at risk. The dynamics of pathogenic *Leptospira* shedding was studied in six dairy herds in southern Chile. Various intermittent urine shedding patterns were found, with elimination periods between 79 and 259 days and bacterial loads ranging from 3 × 10^4^ to 4.4 × 10^4^ bacteria/mL. The current study was the first to assess the various urine shedding patterns and loads of pathogenic leptospires shed through urine of naturally-infected dairy cows. In addition, the study suggests that vaccination does not prevent cattle infection, although it influences loads of pathogenic leptospires excreted in urine. Our study provides a great awareness of asymptomatic animal carriers in an endemic area and will contribute to improving disease control and designing better prevention strategies.

**Supplementary Information:**

The online version contains supplementary material available at 10.1186/s13567-023-01190-w.

## Introduction

Leptospirosis is a zoonotic disease caused by pathogenic spirochetes belonging to the genus *Leptospira*. These disease-causing spirochetes are commonly named pathogenic leptospires to distinguish them from the saprophytic (non-pathogenic) leptospires in the same genus. Domestic and wild animals carry the pathogen in their renal tubules, which act as reservoirs, shedding pathogenic leptospires via their urine to contaminate water resources and soil. In cattle, pathogenic leptospires can also colonize the genital tract [1]. Humans become infected most commonly through contact with the urine of infected animals [1]. Pathogenic leptospires do not survive well in acidic urine but remain viable in alkaline urine. Therefore, herbivores are considered more important shedders than carnivores [2]. Even in the absence of disease, domestic livestock serve as a reservoir for pathogenic leptospires, which represents a significant health risk to a variety of agricultural workers, including farmers, veterinarians and abattoir personnel [2].

During the first 7–10 days after the onset of disease symptoms or signs, pathogenic leptospires can be isolated from blood and cerebrospinal fluid samples. After the second week, following the onset of the disease symptoms or signs, pathogenic leptospires are mainly recovered from urine samples [3]. Persistent colonization of the proximal renal tubules of animal carriers by pathogenic leptospires allows the pathogen to be maintained silently within a host population, especially since infected animals can be asymptomatic while shedding the bacteria in their urine for months [3, 4]. Indeed, experimental infection of cattle has demonstrated that steers begin shedding pathogenic leptospires in their urine between 24 and 30 days after inoculation [5]. Importantly, shedding can extend up to three years, depending on the considered *Leptospira* spp. strain. Shedding can be intermittent or continuous [1] with bacterial loads reaching up to 10^8^ cells per mL in the urine [6]. Chronic pathogenic leptospire carriers play a critical role in maintaining infection within a population [7] by keeping the pathogen circulating in the herd and infecting herd mates. Therefore, detecting and treating carriers is pivotal for an effective control program.

Although the role of cattle as pathogenic leptospire carriers is well recognized, little is known about the extent of their epidemiological importance. The aims of our study were the following: a) to assess the presence and variety of patterns of urine shedding and quantify the load of pathogenic leptospires excreted among naturally-infected cattle; and b) to assess host characteristics that could be associated with those differences in infectiveness. For the first time, this study provides evidence of the various urine shedding patterns and loads of pathogenic leptospires excreted through urine of naturally-infected dairy cows.

## Materials and methods

### Study design and sample collection

A 2-year longitudinal study was conducted from May 2015 to May 2017 on six farms with a history of leptospirosis disease in Chile’s most important dairy regions, Los Lagos and Los Rios. The study was one component of a broader project. It should be noted, five of the six surveyed farms vaccinated their animals against pathogenic leptospires with commercial inactivated vaccines as part of their health programs. In the Chilean market, there are two types of bacterins available: a polyvalent one that includes the *L. borgpetersenii* serovar Hardjo (type Hardjo-Bovis), *L. interrogans* serovar Hardjo (type Hardjo-Prajitno), serovar Pomona, serovar Canicola, serovar Icterohaemorrhagiae, and *L. kirschneri* serovar Grippotyphosa; and a monovalent one that contains *L. borgpetersenii* serovar Hardjo (type Hardjo-Bovis). However, farm records did not specify which type of vaccine was specifically used. Every six months, we obtained a urine sample from all animals in the herd. As these were dairy herds, samples were taken only from female animals. Samples were tested using qPCR (for recruiting new infectious animals from the population). Only those animals that tested positive were followed up on and sampled every 60 days until the end of the trial or the animal left the herd. Manual stimulation of the cow perineal area was practiced for collecting urine in sterile cups. Urine was kept at 4 °C during transport to the laboratory (maximum 2 h).

### Detection of pathogenic leptospires

#### Immunomagnetic-separation (IMS)-coupled lipL32 quantitative polymerase chain reaction

An IMS-qPCR method, previously developed by the study team, that increases the sensitivity of pathogenic leptospires detection from urine samples [8, 9] was used. The method consists of an initial concentration step using polyclonal antibodies coated on magnetic beads prior to qPCR pathogenic leptospire detection [8]. The following steps were then undertaken:

A) At least 1 mL of urine sample was centrifuged at 6500 rpm for 15 min.
B) The remaining pellet was resuspended in 1 mL of 1X phosphate-buffered saline (PBS) and re-centrifuged at 11 000 rpm for 5 min.
C) The supernatant was then discarded, and the pellet was resuspended in 1 mL of 1X PBS [10].
D) After this initial cleaning and purification step, 100 μL of the fluid from Step 3 were separated for a pretreatment step of immune separation using the automated BeadRetrieverTM System (Invitrogen Life Technologies, Grand Island, NY).
E) The final IMS product was suspended in 0.5 mL PBS for DNA extraction-purification (see below).

#### Quantitative real-time lipL32 polymerase chain reaction (qPCR)

Following IMS, each sample was subjected to a DNA extraction-purification protocol using the High Pure PCR Template Preparation kit (Roche), according to the manufacturer’s instructions. Purified DNA was used as a template in a qPCR system (Roche LightCycler 2.0), using a TaqMan probe targeting the *lipL32* gene [10]. The amplification mixture for each sample included 0.5 μL (0.7 μM) of each primer (two primers in total), 0.5 μL (0.15 μM) of probe, 10 μL Master Mix TaqMan universal (Roche), 3.5 μL of water PCR grade (Roche) and 5 μL DNA template, to make a final volume of 20 μL. Samples were amplified with an initial denaturation step at 95 °C for 2 min, followed by 40 cycles of denaturation for 5 s for each cycle at 95 °C and annealing/elongation for 30 s at 58 °C [10, 11]. The positive control for DNA extraction and PCR corresponded to a pure culture of pathogenic leptospires in two dilutions with a known concentration of leptospires (10^4^/mL and 10^2^/mL). Amplification efficiency (E) was calculated from the slope of the standard curve in each run using the formula: E = 10^–1/slope^.

#### Culture

To obtain a culture, 200 μL of urine from each animal were grown in Ellinghausen McCullough, Johnson Harris (EMJH) medium supplemented with 5-fluorouracil (0.2 mg/mL) at 29 °C, according to recommendations by Faine et al. [12]. In summary, each urine sample was first filtered (0.22 µm) and then serially diluted (1:10 and 1:100) in 10 mL of physiological saline solution. Each 1:100 dilution was centrifuged at 3000 x *g* for 10 min, and 200 µL pellets were cultured in liquid EMJH medium and incubated at 29 °C for 100–120 days. The cultures were observed once a week for 13 weeks. DNA was extracted from positive cultures and used for molecular confirmation by qPCR.

#### Serological testing

A microscopic agglutination test (MAT) was performed as reported by Salgado et al. [13], using a panel of six serovars; *L. interrogans* serovars Pomona, Canicola, Icterohaemorrhagiae, Hardjo (type Hardjo-Prajitno) and Autumnalis, and *L. borgpetersenii* serovars Ballum. Serum samples were considered positive when agglutination was observed at titres ≥ 100.

### Estimation of pathogenic *Leptospira* spp. shedding load and shedding time

Pathogenic leptospire cell counts were estimated according to the genome equivalent principle [8] based on the concentration of pathogenic leptospire DNA (from cows with positive qPCR results). The concentration was measured in a Nanoquant spectrophotometer (TECAN group, Männedorf, Schweiz) adjusted for a 10^8^ dilution and the number of copies of the *lipL32* target gene. The genome of *L. interrogans* serovar Hardjo (type Hardjo-Prajitno) was used as the reference of molecular weight (GenBank accession number EU357983.1) and with it a standard curve was established to estimate the pathogenic leptospire numbers in the sample by a Roche 2.0 real-time PCR system, according to a published equation [8].

The estimated total shedding load (Tshed) for each cow was calculated by adding all samples leading to positive qPCR, the product of each urinary load times the number of elimination days, which was calculated as the number of days elapsed between two successive samplings. When two successive samplings yielded only one positive PCR, given that we did not know the exact date of infection and start of shedding, we added half of the days that had elapsed between both samplings.

#### Definitions for urinary patterns of shedding

Animals testing negative for all samples during the follow-up period were labelled as Negative (N). Urinary shedding was classified as Not Persistent (NP) if an animal tested positive by qPCR in a single observation. Persistent (P) animals were those cows testing positive through qPCR in two or more tests. This category was split into two sub-categories based on the length of the shedding period. A Short Persistent (SP) cow was defined as one that had at least two positive qPCR results for at least 90 days. If an animal tested positive for more than 90 days, it was classified as High Persistent (HP). When a negative qPCR alternated in between two positive qPCR results, an animal was classified as Intermittent (I). Multiple Patterns (MP) were defined as animals displaying any other pattern or blended elements of the previously described patterns. Lastly, for each animal, the total number of days shedding (TsheTi) was calculated by adding time intervals between positive and negative qPCR results for each cow.

### Statistical analysis

Descriptive statistics were used to summarize the main parameters. The cow was used as the experimental unit of analysis; however, animals were clustered by farm, therefore this dataset had some amount of hierarchical structure (cow within-farm), therefore, farm was included in the models as a random effect.

A mixed negative binomial regression model accounting for non-negative integers as counts was used to assess differences in total shedding load (Tshed) between shedding patterns, age of the animals and vaccination status. The farm where the animal belonged was used as a random effect in the model. Random effects were used to model many sources of variation as well as subject-specific effects, avoiding biased inference on fixed effects.

We also used an accelerate-to-failure time (AFT) model to assess how the TsheTi could differ between patterns. The survival time logarithms are used as a response variable in this model, along with an error term that is expected to follow a certain probability distribution. The factor $\mathrm{exp}({\beta }_{i}{x}_{i})$ is known as the acceleration factor in the AFT model and is the crucial metric of association as it represents a ratio of survival times that corresponds to a specific value of survival time. In addition, the acceleration factor can be used to assess the impact of predictor variables on survival time; if exp(β_i_*x*_i_) > 1, the covariate effect decelerates it and if exp(β_*i*_*x*_i_) < 1, the covariate effect accelerates it.

The main effects and interactions were then used to build the conditional model using a forward method. The goodness-of-fit model was assessed using the Akaike Information Criteria (AIC) and Bayesian Information Criteria (BIC) indices. For the AFT model, we checked several distributions (Exponential, Weibull, log-normal, log-logistic, Gompertz, gamma and generalized-gamma) and the generalized-gamma distribution was the one with the lower AIC and BIC indices, indicating the best fit.

A *P*-value of 0.05 was used as the threshold for a statistical significance of the differences. All statistics for the negative binomial regression mixed-model were calculated using R’s lme4 package [14]. For the AFT model, the package flexsurv was used for the calculations (V2.1). All data and statistical analysis were processed using R software (version 9.3) [15].

## Results

### Urinary shedding

A total of 194 animals tested positive on at least one of the urinary samples and were thus considered pathogenic leptospire carriers, representing 47.3% of the overall tested population (*n* = 411). No animal showed clinical signs of disease. The percentage of pathogenic leptospire-shedding animals within a herd ranged from 29.5 to 72.7% in the six surveyed farms. Furthermore, animals were distributed over all three considered age groups: 12% among calves (0–12 months), 7% among heifers (> 12–24 months) and 81% among adult cows (> 24 months). Importantly, 64% of pathogenic leptospire-shedding animals were not vaccinated.

Serological testing revealed that 26/194 (13.4%) of the shedders tested positive for MAT. *Leptospira borgpetersenii* serovar Ballum was the most reactive (34.5%) but reactions to *L. interrogans* serovar Hardjo (type Hardjo-Prajitno) (20.7%), *L. interrogans* serovar Autumnalis (17.2%), *L. interrogans* serovar Pomona (13.8%), and less frequently co-agglutinations (10.3%) and *L. interrogans* serovar Canicola (3.4%) were also reported. Concerning the titers, 81.5% presented reciprocal titers of 1/100, and the other 18.5% ranged between 1/200 and 1/400.

**Figure 1:**
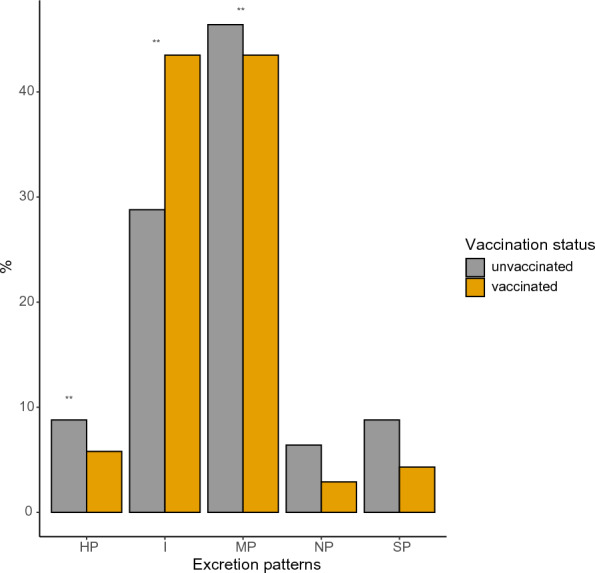
**Distribution of urine shedding patterns in dairy cows from Chile, for non-vaccinated and vaccinated herds**. %: is percentage of leptospirosis urine-shedders animals. Definitions: Not Persistent (NP); Short Persistent (SP); High Persistent (HP); Intermittent (I); Multiple Patterns (MP). ** means that there is a statistically significant (P<0.05) difference between vaccinated vs non-vaccinated group for a given pattern.

Among PCR-positive animals, MP was found in 45% of the animals (95% CI 38.4; 52.4) and IP in 34.0% (95% CI 27.4; 40.7). HP, SP and NP were found in 7.7% (95% CI 4.0; 11.5), 7.2% (95% CI 3.6; 10.9), and 5.2% (95% CI 2.0; 8.3) of positive animals, respectively. SP pattern was seen more frequently in calves (25%) than in cows (5%), and the difference was statistically significant. On the contrary, HP was detected more often in heifers than in cows (31% vs. 7%, *p* < 0.05). The frequency of IP and MP was similar in all three groups (*p* > 0.05). Vaccinated cattle had a larger proportion of individuals with IP compared to unvaccinated cattle, but all other patterns (NP, SP, HP, and MP) were found with similar frequencies in vaccinated and unvaccinated animals (Figure 1).

Table 1 shows that HP, IP, and MP patterns have the longest shedding periods (272 to 395 days), and that the differences between them were statistically significant (they revealed, for example, a deacceleration factor (> 1)). For the shortest patterns, time shedding differs between vaccinated and non-vaccinated animals (SP and HP). However, given a substantially greater AIC and BIC, the goodness-of-fit (GOF) of the model that incorporated interaction with vaccination status suggests that this interaction did not increase GOF. Furthermore, the AFT result demonstrates a statistically significant difference in the shedding periods of vaccinated and unvaccinated animals (Table 2).

**Table 1:** **Descriptive statistics for the pathogenic *Leptospira* spp. shedding time (in days) for the different urine patterns; age group and for vaccinated and unvaccinated animals; obtained from dairy cattle in southern Chile.**

Variable	Category	n	Overall		Unvaccinated		Vaccinated	
			Mean (SD)	Median (IQR)	Mean (SD)	Median (IQR)	Mean (SD)	Median (IQR)
Shedding pattern	*NP*	10	122 (68)	134 (82)	143 (69)	147 (36)	NC	NC
	*SP*	14	93 (82)	62 (67)	126 (70)	111 (69)	81 (90)	53 (51)
	*HP*	15	272 (95)	255 (78)	221 (46)	206 (48)	292 (107)	258 (141)
	*I*	66	358 (134)	394 (183)	351 (145)	358 (232)	362 (128)	400 (106)
	*MP*	88	395 (109)	409 (142)	398 (108)	411 (170)	391 (109)	402 (133)
Age group	*Young stock*	25	203 (136)	217 (289)	203 (136)	217 (289)	NC	NC
	*Adult*	168	356 (139)	381 (196)	368 (131)	400 (165)	355 (137)	355 (204)
Vaccination status*	*Unvaccinated*	119	333 (148)	355 (194)	NC	NC	NC	NC
	*Vaccinated*	68	355 (138)	355 (203)	NC	NC	NC	NC

NP: not persistent; SP: short persistent (< 90 days); HP: high persistent (> 90 days); I: Intermittent; MP: Multiple Pattern; NC: not calculated (because there were no animals falling in this category); *there are missing values in the variable.

**Table 2:** **Accelerate-to-failure time final model estimations for the pathogenic *Leptospira* spp. shedding time obtained from dairy cattle in southern Chile.**

Variable	Category	Exponential of estimate	95%CI
Shedding pattern	*NP*	0.44*	0.37; 0.51
	*SP*	0.69*	0.52; 0.92
	*HP*	Ref.	
	*I*	2.12*	1.55; 2.92
	*MP*	2.78*	1.90; 4.05
Vaccination status	*Unvaccinated*	Ref.	
	*Vaccinated*	0.54*	0.42; 0.69

NP: not persistent; SP: short persistent (< 90 days); HP: high persistent (> 90 days); I: intermittent; MP: multiple pattern; * *P*-value < 0.05; AIC: 934.61; BIC = 957.22.

### Bacterial burden for urine shedding pattern

**Figure 2:**
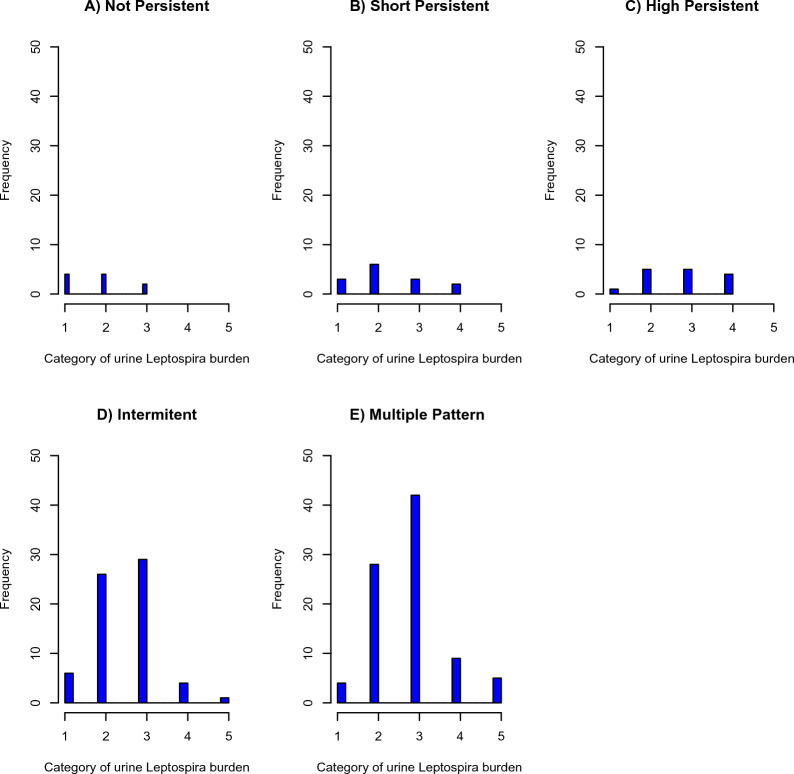
**Distribution of pathogenic leptospire burden (GE/μL) according to different shedding patterns; data obtained from dairy cattle in southern Chile**. Group 1: < 1000 (GE/μL), group 2: 1000 to < 10^3^ (GE/μL), group 3: 10^3^ to < 10^4^ (GE/μL), group 4: 10^4^ to 10^5^ (GE/μL), group 5: > 10^5^ (GE/μL).

**Figure 3:**
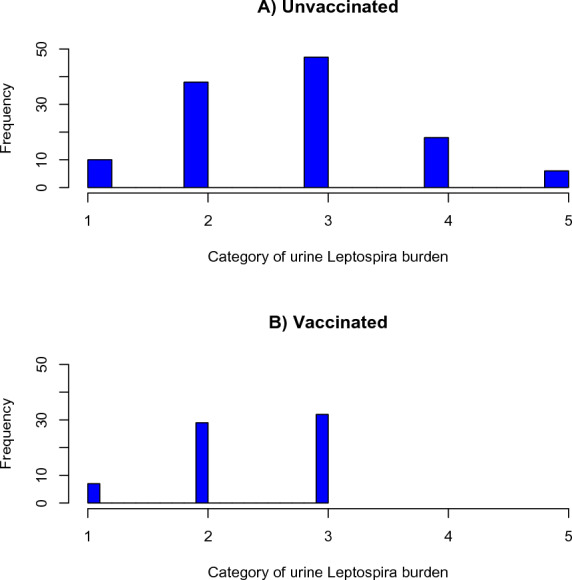
**Distribution of pathogenic leptospire burden (GE/μL) according to vaccination status of the animals; data obtained from dairy cattle in southern Chile.** Group 1: < 1000 (GE/μL), group 2: 1000 to < 10^3^ (GE/μL), group 3: 10^3^ to < 10^4^ (GE/μL), group 4: 10^4^ to 10^5^ (GE/μL), group 5: > 10^5^ (GE/μL).

Additional file 1 summarizes the estimated mean and median urine excretion loads for the various urine patterns and attributes of the animals (age group and vaccination status). In general, there are wide discrepancies between means and medians, indicating non-symmetrical distributions and the presence of outliers (Figures 2 and 3), particularly among individuals that excrete heavy bacterial loads and represented by groups 4 and 5.

The Negative-Binomial final model shows a statistically significant difference (*P* < 0.05) in the number of pathogenic leptospires excreted in the urine of animals with shorter shedding patterns (NP and SP) than in HP animals: bacterial loads in NP and SP are 0.19 and 0.24 times of the HP loads. Interestingly, no statistically significant difference (*P* > 0.05) was found in the number of pathogenic leptospires excreted in the urine of unvaccinated and vaccinated animals (Table 3).

**Table 3:** **Negative binomial final model for pathogenic leptospire urine elimination load (GE/mL) obtained from dairy cattle in southern Chile.**

Variable	Category	Estimation	95% CI	*p*-value
Shedding pattern	*NP*	0.188	0.050; 0.868	0.019
	*SP*	0.241	0.062; 0.947	0.024
	*HP*	Ref.		
	*I*	0.483	0.168; 1.231	0.125
	*MP*	0.703	0.243; 1.809	0.447
Vaccination status	*Non vaccinated*	Ref.		
	*Vaccinated*	1.353	0.589; 2.863	0.422

NP: not persistent SP: short persistent (< 90 days); HP: high persistent (> 90 days); I: intermittent; MP: multiple pattern; $\theta$= 0.4473; AIC = 4372.1; BIC = 4410.9.

## Discussion

The current study reports a high prevalence of carriers with no clinical signs of leptospirosis among cattle in Chile. These numbers are still lower than those reported in studies carried out in Brazil, where 63.9% [16] and 68.4% [17] of tested animals were actually reported as carriers. Notably, these studies were either based on cows sampled at slaughterhouses or on animals with reproductive disorders, which is not comparable to the sample tested in our study.

One of the most significant study results is the range and complexity of shedding patterns displayed by bovine carriers. Many bovines eliminating pathogenic leptospires do so in a variety of ways, the most frequently through pattern categories that we describe herein as multiple and intermittent. Although many studies have previously investigated urine shedding in bovines, such studies either followed up animals for shorter periods or used other diagnostic methods for detecting pathogenic leptospires in urine. Rocha et al. [18] used a conventional PCR allowing an intermittent pattern to be reported but animals were sampled weekly for only six weeks. Furthermore, a typical pattern was mentioned but not comprehensively defined, which did not allow their findings to the figures reported herein to be compared. Another study [5] reported a light intermittency in steers experimentally infected with *L. interrogans* serovar Hardjo (type Hardjoprajitno) using another technique (culture and dark-field microscope) in a survey lasting 52 days. A study using 20 experimentally infected Friesian heifers reported a constant although declining shedding for up to 60 weeks [4]. Animals were examined for 22 months using the same diagnostic approach described by Sullivan [5] and experimentally infecting cattle with *L. interrogans*, serovar Hardjo (type Hardjoprajitno), and *L.* *borgpetersenii* Hardjo (type Hardjobovis). Gerritsen et al. [19] observed in the non-treated group a continuous excretion pattern in cows experimentally infected with the same pathogenic leptospire strain as reported in [4]. This study detected leptospiruria using PCR in urine sampled every week for six months, with shedding periods ranging from 8 to 22 weeks.

A critical difference with the studies mentioned above is that these were not based on natural infection conditions since animals were experimentally exposed to one variant or serovar. Non-adapted pathogenic leptospire strains may influence the intermittency pattern by using cattle as an accidental host. This characteristic is strengthened by Monahan et al. [20], who reported that urine excretion in accidental hosts is low, intermittent, and brief. Serovar Hardjo has been stated as adapted to the bovine species [2]. We can expect diverse excretion patterns when different strains of pathogenic leptospire serovar Hardjo are used in experiments. However, it is uncertain whether the isolates used in these studies belong to one or two *Leptospira* species associated with the Hardjo serovar (*L. interrogans*, *L. borgpetersenii*). As a result, future research might compare and examine both species to see if there are any variations in infected cattle’s urine elimination patterns.

Our findings highlight important information for designing new or adjusting current control strategies. The intermittent pattern detected by PCR in naturally-infected animals demonstrates how difficult it is to identify infected animals with a single sample. As a result, an accurate control measure requires more than one sample per animal. In addition, a control program will be more successful if using PCR on urine samples instead of relying on serological results, given that many carriers and shedders will be not detected through serology. We isolated pathogenic leptospires from the urine of seronegative animals, confirming that serology is not relevant to identify the pathogenic leptospire carrier status in cattle [16], as reported for other wild reservoir animals [21].

With a mean elimination of 259 days, ranging from 207 to 311 days, the animals with MP and HP patterns excreted pathogenic leptospires for a longer period of time than animals with SP. This length in time is similar to that reported in a previous study [22], estimating the shedding time length to an average of 182 days (ranging from 56 to 378 days) in intrauterine infected heifers and 224 days (ranging from 84 to 420 days) for the supra conjunctival infection route, although the different shedding patterns were not determined in these experimental infections using *L. borgpetersenii* serovar Hardjo (type Hardjobovis). This difference could be due to the experimental infection method that was used even though a previous study from the same group [4] reported a broad range of days for bacteria to be eliminated (larger than 280 days) [22].

The median rate in naturally-infected animals in our study ranged from 3.4 × 10^3^ GE/mL to 22.9 × 10^3^ GE/mL. Gerritsen et al. showed that calves experimentally infected with *L.* *borgpetersenii* serovar Hardjo (type Hardjobovis) strain via the intraocular route have a 10^3^–10^5^ /mL burden, as measured by conventional PCR [19]. However, because our estimates were based on natural infections, the loads were likely caused by a variety of unidentified infection pathways, given the observational designs used. In addition, Monahan et al. [20] claimed that strains that were not adapted to the animals’ species resulted in low elimination intensity in the affected animals.

Despite this, the number of bacteria excreted in urine highlights the importance of bovines in shedding pathogenic microorganisms in the environment. Although the estimated bacterial load is much lower than that reported in other animal species, such as brown rats in Brazil with loads ranging from 2.2 to 0.4 × 10^6^ GE/mL [23] and dogs with a burden of 35.5–1.33 × 10^6^ GE/mL [24], bovines should be considered a significant source of environmental contamination given the volume of urine eliminated per day [6].

Another noteworthy finding is the variety in urine rates, particularly the significantly large ones, which indicates that some animals might be more infectious than others. Even though many infectious diseases have been described as having super-spreaders [25], our findings are insufficient to reach a conclusion.

A statistically significant difference in shedding rates was also found between patterns with a larger amount and longer excretion time. This result is also critical for the development of improved control strategies, emphasizing the need of recognizing those infected animals early.

Although the purpose of this study was not to compare unvaccinated and vaccinated animals, preliminary findings on pattern and elimination rates for vaccinated and unvaccinated animals are striking. We report pathogenic leptospire shedding in both vaccinated and unvaccinated animals. Shedding was significantly shorter in vaccinated animals (Table 2) although there was no statistically significant difference in the bacterial loads between the two groups (Table 3). Commercial vaccines (either monovalent or multivalent) that are currently available have been shown to be effective at preventing clinical illness (such as reproductive failure), but they do not offer immunity (such as the ability to stop urine shedding) or protection against different leptospira serovars [26, 27]. In addition, age at first vaccination may be significant, particularly in different farm settings, because vaccination is less effective in reducing urinary shedding in infected animals than in naive animals [28]. Vaccinated animals presented more IP patterns than unvaccinated animals. Although intermittent urine shedding patterns have been reported, our findings suggest that this association with vaccination status warrants further investigation. Altogether, our findings suggest that immunization does not significantly prevent the prevalence of carriers nor influence shedding characteristics. However, the animals in this study had a wide variety of vaccination periods, with some being vaccinated for up to 6 months afterward, showing that vaccination alone does not protect the herd from further transmission.

The current study is the first to assess the various urine shedding patterns and loads of pathogenic leptospires eliminated through urine of naturally-infected dairy cows. In addition, the study suggests that vaccination does not prevent cattle infection, although it influences the load of pathogenic leptospires shed in urine. Our study provides a great awareness of asymptomatic animal carriers in an endemic area and will contribute to improving disease control and designing better prevention strategies.

## Supplementary Information

Additional file 1. Descriptive statistics for the pathogenic *Leptospira *spp. urine load (GE/mL * 10^**3**^**) by different urine patterns, and characteristics of the animals (age group and vaccination status); obtained from dairy cattle in southern Chile.**

## Data Availability

The datasets used during the current study are available from the corresponding author on reasonable request.
