# Supplementary material for: Urine shedding patterns of pathogenic Leptospira spp. in dairy cows
Source: Vet Res. 2023 Jul 31;54:64. doi: 10.1186/s13567-023-01190-w (PMC10391894; doi:10.1186/s13567-023-01190-w)
Supplement: Supplementary file 1 — Additional file 1. Descriptive statistics for the pathogenic Leptospira spp. urine load (GE/mL * 103) by different urine patterns, and characteristics of the animals (age group and vaccination status); obtained from dairy cattle in southern Chile. [file 13567_2023_1190_MOESM1_ESM.docx]

|  |  |  | **Overall** | | **Unvaccinated** | | | **Vaccinated** | | | |  |
| --- | --- | --- | --- | --- | --- | --- | --- | --- | --- | --- | --- | --- |
| **Variable** | **Category** | **n** | **Mean**  **(SD)** | **Median**  **(IQR)** |  | **Mean**  **(SD)** | **Median**  **(IQR)** | |  | **Mean**  **(SD)** | **Median**  **(IQR)** | |
| Shedding pattern | *NP* | 10 | 16.3  (30.5) | 3.4  (7.0) |  | 15.8  (34.3) | 2.5  (6.4) | |  | NC | NC | |
|  | *SP* | 14 | 36.8  (83.7) | 3.6  (17.2) |  | 37.1  (95.6) | 2.7  (10.3) | |  | 9.3 (9.7) | 4.6  (8.8) | |
|  | *HP* | 15 | 92.4  (1940.3) | 22.9  (96.2) |  | 127.6  (233.0) | 15.5  (150.3) | |  | 21.6 (20.3) | 18.7  (21.4) | |
|  | *I* | 66 | 114.1  (692.6) | 10.3  (26.1) |  | 195.3  (935.8) | 13.9  (49.2) | |  | 16.7  (22.6) | 6.5  (19.3) | |
|  | *MP* | 88 | 916.1  (6226.4) | 14.4  (50.5) |  | 1429.2  (7783.8) | 25.3  (84.3) | |  | 18.8  (24.2) | 11.5  (16.6) | |
| Age group | *Youngstock* | 25 | 22.7  (62.2) | 3.2  (11.5) |  | 22.8  (62.3) | 3.3  (11.6) | |  | NC | NC | |
|  | *Adult* | 168 | 533.6  (4533.0) | 13.6  (48.1) |  | 938.9  (6043.4) | 23.0  (85.6) | |  | 17.7  (22.7) | 9.3  (17.4) | |
| Vaccination status* | *Unvaccinated* | 119 | 17.6  (22.6) | 9.2  (17.3) |  | NC | NC | |  | NC | NC | |
|  | *Vaccinated* | 68 | 746.4  (5378.2) | 13.9  (57.8) |  | NC | NC | |  | NC | NC | |

NP: Not Persistent; SP: Short Persistent (<90 days); HP: High Persistent (>90 days); I: Intermittent; MP: Multiple Pattern; NC= not calculated (because there were no animals falling in this category); * there are missing values in the variable
